# Comparison of buccal and blood-derived canine DNA, either native or whole genome amplified, for array-based genome-wide association studies

**DOI:** 10.1186/1756-0500-4-226

**Published:** 2011-06-30

**Authors:** Gonzalo Rincon, Katarina Tengvall, Janelle M Belanger, Laetitia Lagoutte, Juan F Medrano, Catherine André, Anne Thomas, Cynthia Taylor Lawley, Mark ST Hansen, Kerstin Lindblad-Toh, Anita M Oberbauer

**Affiliations:** 1 Department of Animal Science, University of California, Davis, CA 95616, USA; 2 Science for Life Laboratory, Department of Medical Biochemistry and Microbiology, Uppsala University, Uppsala, Sweden; 3 UMR6061 Institut de Génétique et Développement, Centre National de la Recherche Scientifique, Université de Rennes 1, F-35000 Rennes, France; 4 Antagene, Animal Genetics Laboratory, 2 Allée des Séquoias, 69578, Limonest (Lyon), France; 5 Illumina, Inc. 9885 Towne Centre Drive, San Diego, CA 92121, USA; 6 The Broad Institute, Seven Cambridge Center, Cambridge, MA 02142, USA

## Abstract

**Background:**

The availability of array-based genotyping platforms for single nucleotide polymorphisms (SNPs) for the canine genome has expanded the opportunities to undertake genome-wide association (GWA) studies to identify the genetic basis for *Mendelian *and complex traits. Whole blood as the source of high quality DNA is undisputed but often proves impractical for collection of the large numbers of samples necessary to discover the loci underlying complex traits. Further, many countries prohibit the collection of blood from dogs unless medically necessary thereby restricting access to critical control samples from healthy dogs. Alternate sources of DNA, typically from buccal cytobrush extractions, while convenient, have been suggested to have low yield and perform poorly in GWA. Yet buccal cytobrushes provide a cost-effective means of collecting DNA, are readily accepted by dog owners, and represent a large resource base in many canine genetics laboratories. To increase the DNA quantities, whole genome amplification (WGA) can be performed. Thus, the present study assessed the utility of buccal-derived DNA as well as whole genome amplification in comparison to blood samples for use on the most recent iteration of the canine HD SNP array (Illumina).

**Findings:**

In both buccal and blood samples, whether whole genome amplified or not, 97% of the samples had SNP call rates in excess of 80% indicating that the vast majority of the SNPs would be suitable to perform association studies regardless of the DNA source. Similarly, there were no significant differences in marker intensity measurements between buccal and blood samples for copy number variations (CNV) analysis.

**Conclusions:**

All DNA samples assayed, buccal or blood, native or whole genome amplified, are appropriate for use in array-based genome-wide association studies. The concordance between subsets of dogs for which both buccal and blood samples, or those samples whole genome amplified, was shown to average >99%. Thus, the two DNA sources were comparable in the generation of SNP genotypes and intensity values to estimate structural variation indicating the utility for the use of buccal cytobrush samples and the reliability of whole genome amplification for genome-wide association and CNV studies.

## Findings

The present study was undertaken to assess the utility of buccal cytobrush derived DNA and whole genome amplified (WGA) blood or buccal-derived DNA for use on the most recent iteration of the canine SNP GWA platform. Buccal-derived DNA has been suggested as insufficient in quantity and quality for application to the high-throughput SNP array platforms [1]. Whole blood DNA and buccal-derived DNA, as well as DNA samples (from both sources) subjected to WGA, were compared using the Illumina Infinium CanineHD Genotyping BeadChip containing 173,662 SNPs. Copy number variations (CNV), while shown to account for a significant proportion of human genetic polymorphism and have been suggested to play a role in genetic causes of disease [2], is complex and technically challenging to analyze. Specifically CNV analysis is uniquely different to GWA-SNP analysis because the data is based on the intensity measurement of the SNP. Despite the technical issues, the opportunity exists to examine this important feature of the genome using high quality tools like the canine Illumina HD chip. In this study we developed an expanded comparative study using intensity files to test whether buccal cytobrush derived DNA would affect CNV segment results in the Illumina Infinium CanineHD Genotyping BeadChip.

## Materials and methods

### Samples

To assess concordance between the array performance for buccal and blood samples, both blood and buccal samples were collected for eight Bearded collies. To evaluate genomic DNA preparation using WGA, DNA samples from an additional nine dogs from five breeds were used for comparison between native blood, blood WGA, and buccal WGA DNA. In addition, a larger sample size where either buccal cytobrush (82) or blood samples (146) were collected from 228 Standard poodles in Europe and in the United States as part of our ongoing studies to identify the genetic basis for hypoadrenocorticism. All animal work was approved by the University of California, Davis Institutional Animal Care and Use Committee or by the Ethical board for experimental animals in Uppsala, Sweden (C139/9) or by the CNRS ethical board approval, France (35-238-13) and samples were voluntarily submitted by private dog owners.

### DNA Extraction

Buccal-derived DNA was extracted as previously reported [3]. For the WGA comparisons, genomic DNA was extracted from buccal samples preserved in ethanol using the NucleoSpin 96 Tissue DNA Kit (NucleoSpin 96 Tissue DNA kit, Macherey Nagel, Hoerdt, France) according to the manufacturer's instructions. Blood samples (200 μl) were extracted using the QIAGEN QIAamp^® ^DNA Blood Mini and Midi Kits (QIAGEN Inc., Valencia, CA) or the Nucleospin kit (Machery Nagel). Extracted DNA was stored at -20/-80°C until use. Quantification of the extracted DNA samples was performed using the NanoDrop^® ^(ND-1000 v3.2.1) spectrophotometer (Thermo Scientific, Wilmington, DE). Eight Bearded collie buccal-derived DNA samples with eight matching blood-derived DNA samples were evaluated on the SNP arrays. Samples were from dogs of all ages, both sexes (5 males, 3 females), and the assayed DNA was either freshly extracted or stored for up to 6 years. Standard poodle samples were blood or buccal from dogs of all ages, both sexes (82 males, 132 females, and 14 of unknown sex), and the assayed DNA was either freshly extracted or stored for up to 6 years. For an additional nine dogs from five breeds (5 males, 4 females), native blood-derived DNA was compared to the matching blood-derived DNA samples and buccal-derived DNA samples subjected to WGA as per manufacturer's instructions (Genomeplex complete WGA 2 kit, Sigma, Missouri USA).

### Genotyping

Samples were genotyped using the Illumina Infinium CanineHD Genotyping BeadChip (Illumina Inc. San Diego, CA) by Geneseek (Lincoln, NE). Illumina's GenCall algorithm was used to call genotypes (Illumina Inc. San Diego, CA).

### Analyses

The software package PLINK v.1.06 [4] was used to calculate call rates. The option--missing was used to calculate the frequency of missing SNPs per sample. From this data, genotype call rates could be calculated for each sample. The data were analyzed both with and without quality control criteria. Quality control criteria (filters) were used to remove from further analysis any individual sample having less than 10% of all SNPs genotyped, an overall amplification for a given SNP of 90%, and a minor allele frequency of 0.01.

Basic genotype statistics for each marker, including call rate, minor allele frequency, Hardy-Weinberg Equilibrium (HWE) P-value, Correlation R, and allele and genotype counts were calculated using the "Quality Assurance Module" from SNP Variation Suite version 7 (SVS7) (Golden Helix Inc., Bozeman, Montana, USA).

CNV analysis was performed to define regions of CNV on a genome-wide scale, sample by sample (univariate analysis) with the copy number analysis module (CNAM) from SVS7. The signal intensity files for each SNP (log 2 ratio data) and the genetic marker map were downloaded with a custom SVS7 script from Illumina Genome Studio. To normalize data, principle component analysis was performed on the intensity data to correct for error/chip variation for each sample. CNV segments were defined using a moving window of 5000 SNPs, with 20 segments per window and a minimum number of one SNP per segment. A linear regression was performed to test for differences in CNV segments obtained in the eight Bearded collie buccal-derived DNA samples and the eight matching blood-derived DNA samples. In addition, CNV segments were analyzed for the WGA samples to assess whether the amplification influenced detection of CNV when compared to native blood-derived DNA.

## Results

### DNA sample yield

For Bearded collies, the average concentration obtained for buccal-derived DNA (n = 8) was 83.14 ng/μl and that for blood-derived DNA (n = 8) was 46.44 ng/μl. For Standard poodles, the average concentration obtained for buccal-derived DNA (n = 82) was 120.4 ng/μl and that for blood-derived DNA (n = 146) was 53.2 ng/μl. For the dogs for which native blood, WGA blood-derived, and WGA buccal-derived DNA were prepared, average yields were 70 ng/μl for blood-derived DNA and 25 ng/μl for buccal-derived DNA.

### Genotyping Call Rates (PLINK)

Across all samples analyzed, 1547 SNPs failed to genotype resulting in 172,115 available SNPs for analysis. The average call rate of the eight Bearded collies with paired buccal and blood samples was assessed. Blood samples had an average call rate of >99.6% (range 99.6% to 99.7%) while the buccal samples had an average call rate of 98.7% (range 95.7% to 99.6%). For the eight Bearded collie samples, the concordance in SNP calls between the buccal and blood was >99.16% on average (range 95.69% to 99.72%). Lower concordance was observed for samples that had the greatest number of SNPs that failed to be genotyped (no calls). If SNPs that failed to genotype for a given sample were omitted, then the concordance in SNP calls between blood and buccal increased to an average of >99.91% (range 99.65% to 99.97%). Thus, mismatched calls represented 0.09% of discordance while SNPs with no calls represented 0.84%. For the Standard poodles (n = 228) overall genotyping call rates for blood versus buccal are presented in Table 1. Call rates for data with no SNPs filtered were 98.46% for blood and 97.71% for buccal. Post-filter call rates were 98.46% for blood and 97.81% for buccal. The post-filter call rate for buccal increased slightly based upon the removal of a single sample that was removed for low genotyping (<90%) based on the quality control criteria.

**Table 1:** Call rates for blood and buccal cytobrush samples (total number of SNPs = 173,662) as determined using SVS7 and PLINK software programs.

	N	Mean Call Rate	SNP with Call Rate = 0	SNP with Call Rate <0.5	SNP with Call Rate >0.5 < 0.8	SNP with Call Rate >0.8
**SVS7**						
Blood	146	0.98 ± 0.10	1547 (0.9%)	279 (0.16%)	1101 (0.63%)	170735 (98.3%)
Buccal	82	0.97 ± 0.11	1548 (0.9%)	672 (0.38%)	2773 (1.60%)	168669 (97.1%)
						
**PLINK**						
Blood	146	0.98 ± 0.04	1547 (0.9%)	257 (0.15%)	1152 (0.66%)	170706 (98.3%)
Buccal	82	0.97 ± 0.14	1548 (0.9%)	635 (0.37%)	2789 (1.61%)	168690 (97.1%)

Genotyping call rates were unaffected by the duration of DNA storage. Call rates were assessed using PLINK without any quality control criteria. Of the 82 Standard poodle buccal samples, 10 were processed within 24 months of the SNP assay and 72 were stored between 24 and 72 months prior to assay. The call rate for the samples assayed within 24 months of collection was 97.62% and the call rate for the older samples was 97.72%. There was no difference (p > 0.05) for buccal sample genotype call rates based on duration of sample storage.

In the analysis of the concordance for the WGA samples relative to the native blood-derived DNA, the native blood samples had an average call rate of >99.8%, the WGA blood samples had an average call rate of >99.3%, while the WGA buccal samples had an average call rate of 98.9%. For true mismatched calls, that is with SNPs that failed to genotype omitted, the concordance in SNP calls between the WGA buccal samples and WGA blood samples with native blood was >99.984% on average (0.016% discrepancy). The concordance between WGA buccal and WGA blood was >99.988% (0.012% discrepancy). See Table 2 for average values per breed. Similar to the findings above, of the discordance, SNPs with no calls represented 0.846% for WGA buccal compared to native blood, 0.886% for WGA blood compared to native blood, and 0.993% for WGA buccal compared to WGA blood.

**Table 2:** Comparison of three DNA sources (native blood, WGA blood-derived DNA and WGA buccal-derived DNA) on SNP differences observed after genotyping nine dogs on the canine HD SNP array (Illumina).

	Total number of dogs	Average number of SNP differences per dog		
		**Native Blood/WGA Blood**	**Native Blood/WGA buccal**	**WGABlood/WGA buccal**

SNP discrepancies	9	21 SNPs	37 SNPs	20 SNPs

Average differences between SNPs	9	(0.012%)	(0.020%)	0.012%

Average concordances between SNPs	9	99.988%	99.980%	99.988%

English Bull terrier	4	24 SNPs	42 SNPs	18 SNPs

Czechoslovakian wolf dog	1	9 SNPs	34 SNPs	41 SNPs

Tibetan terrier	1	63 SNPs	39 SNPs	15 SNPs

Yorkshire terrier	1	11 SNPs	41 SNPs	28 SNPs

Standard poodles	2	4 SNPs	29 SNPs	12 SNPs

For four dogs representing two breeds, the native blood, WGA blood, and WGA buccal samples were assayed in duplicate and the genotypes compared between the duplicates. One duplicate showed 34 SNP differences for true mismatched calls but for the remaining duplicated samples, there were no differences in genotypes out of the 173,662 SNP markers assessed. In addition, among SNPs with a discordant genotype between the three DNA preparations, 19 SNPs were involved more than ten times, indicating that the discrepancies are most probably due to SNPs that proved difficult to genotype and not the DNA quality. Genotypes for samples from the same DNA source and preparation, when assayed in duplicate, yielded average call rates of 99.8% and average concordance of 99.97%. Hence the genotype variation observed for DNA from different sources or preparation was not greatly different from that observed for a single sample assayed in duplicate.

### Genotype Statistics by Marker and Sample (SVS7)

To assess quality of data derived from the blood and buccal Standard poodle samples, basic genotype statistics for each marker, were also calculated using the SVS7 software program (Table 1). The correlation between call rates for blood/buccal was r = 0.95. The average call rate for the 173,662 SNPs surveyed was 0.98 ± 0.10 for blood and 0.97 ± 0.11 for buccal.

**Figure 1:**
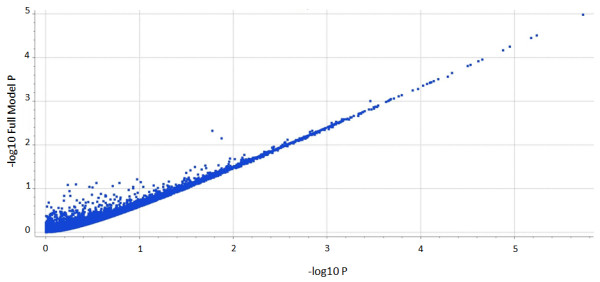
**Plot of full vs. reduced model (-log10 P values)**. X-axis reduced model including DNA source as covariate. Y-axis full model. 173,662 markers were sorted by (-log10 P values). The r = 0.95 indicates the equivalence of the buccal and blood derived DNA in the generation of SNP genotypes.

To address the question of whether the DNA source (blood versus buccal) might obfuscate interpretation of association with a particular disease, the response to a linear regression using a full versus reduced model was applied considering disease status of the samples. Specifically, the linear regression analysis was included to demonstrate that the data derived from the different sources had equivalent utility in an actual analysis of 228 Standard poodle DNA samples from cases (70 blood, 42 buccal) and controls (76 blood, 40 buccal) for hypoadrenocorticism. To do this, first a linear regression equation, which included only the dependent and the reduced model covariate (blood/buccal), was calculated ("reduced model"). Next, a linear regression which included all variables (sex, DNA source, country, disease status) was calculated ("full model"). The significance of the full versus the reduced model was calculated with an F-test (p = 0.51). Figure 1 shows the results from the full vs. reduced model regression and illustrates the performance equivalence of the blood and buccal samples in the assay.

### Copy Number Variation

Segments (6,664 segments distributed across the genome) were defined for the Bearded collie buccal and blood samples. Using CNV segments on chromosomal regions the CNV association analysis to examine structural variation in the canine genome showed no significant differences in CNV segmentation and marker intensity measurements between buccal cytobrush and blood samples (p > 0.2). Similarly, segments (7,200) defined for the WGA samples, buccal and blood, were compared to native blood. Again no significant differences were detected (p > 0.8).

## Discussion

Buccal cytobrush collections offer a simple non-invasive means of DNA collection. Concern over efficiency of buccal-derived DNA for GWA SNP platforms has focused upon yield and purity [1], in particular contamination from resident microbes within the oral cavity. However, the utility of buccal cytobrush extracted DNA that was whole-genome amplified was demonstrated for a small scale, custom, single chromosome, canine SNP array [5] and also for human array genotyping [6]. Saliva- derived DNA has been reported as an alternate source of DNA for high-quality data for use in GWA studies though the sample size was small and the array carried 22,362 SNPs and microbial contamination remains a concern. In dog saliva sampling, bacterial DNA contamination has been reported to be 16.1% [7]. Woo et al., [6] and Yokoyama et al., [1] considered bacterial contamination to be insignificant based on the concordance of the samples and the high call rates for buccal samples and saliva samples, respectively. The findings of the current study support the view that oral bacterial DNA contamination is minor.

The present study is the first to evaluate the efficacy of buccal DNA for large-scale GWA studies directly comparing to blood using a significant sample size. In both buccal and blood samples 97% of the samples had SNP call rates in excess of 80% indicating that the vast majority of the SNPs would be suitable to perform association studies regardless of the DNA source. Results from the association study were not affected when DNA source was included in the analysis. Further, the concordance between a subset of eight Bearded collies for which both buccal and blood samples were analyzed averaged >99%. When considering the need to subject samples to WGA prior to genotyping on the SNP array, the average SNP call rates showed that native blood samples (>99.8%) > WGA blood samples (>99.3%) >> WGA buccal samples (98.9%). The concordance in SNP calls between the native blood and the WGA blood samples was 99.988% and the concordance between the native blood and the WGA buccal samples was 99.980%. The concordance between the average of WGA (buccal and blood samples) with native blood was >99.984% (Table 2). These comparisons made on nine dogs from five different breeds showed that the concordance of genotyped SNPs is excellent between native blood and WGA blood samples, indicating that when needed, WGA can be performed with confidence (0.012% discrepancies). For buccal samples, there is a slight improvement with WGA (>99.91% and 99.98%, for buccal and WGA buccal concordance with native blood). Thus, native and WGA buccal and blood-derived DNA generated comparable SNP genotypes indicating the utility for the use of stored buccal cytobrush samples for genome-wide association and CNV studies.

## Competing interests

The authors declare that they have no competing interests.

## Authors' contributions

GR carried out the CNV and SVS7 analyses and helped draft the manuscript; KT participated in the study design and coordination, helped draft the manuscript, performed sample collection and data analysis; JMB participated in study design, performed PLINK and helped perform the SVS7 analyses, coordinated sample collection, and helped draft the manuscript; LL provided input on study design and WGA analysis and coordinated sample collection; JFM provided input in study design, analysis, and drafting the manuscript; CA provided input on study design, analysis, and helped draft the manuscript; AT identified matched provided buccal cytobrush and blood samples and participated in the analysis; CTL participated in study design, performed the WGA statistical analyses, and helped draft the manuscript; MSTH assisted in study design and the WGA studies; KLT participated in study design, analysis, study coordination, and drafting the manuscript; AMO participated in study design, analysis, study coordination, and drafting the manuscript. All authors have read and approved the final manuscript.
